# The impact of read length on quantification of differentially expressed genes and splice junction detection

**DOI:** 10.1186/s13059-015-0697-y

**Published:** 2015-06-23

**Authors:** Sagar Chhangawala, Gabe Rudy, Christopher E. Mason, Jeffrey A. Rosenfeld

**Affiliations:** The Institute for Computational Biomedicine, Weill Cornell Medical College, New York, NY 10021 USA; Department of Physiology and Biophysics, Weill Cornell Medical College, New York, NY 10021 USA; Golden Helix, 203 Enterprise Blvd, Suite 1, Bozeman, MT 59718 USA; Rutgers-Cancer Institute of New Jersey, New Brunswick, NJ 08901 USA; American Museum of Natural History, New York, NY USA

## Abstract

**Background:**

The initial next-generation sequencing technologies produced reads of 25 or 36 bp, and only from a single-end of the library sequence. Currently, it is possible to reliably produce 300 bp paired-end sequences for RNA expression analysis. While read lengths have consistently increased, people have assumed that longer reads are more informative and that paired-end reads produce better results than single-end reads. We used paired-end 101 bp reads and trimmed them to simulate different read lengths, and also separated the pairs to produce single-end reads. For each read length and paired status, we evaluated differential expression levels between two standard samples and compared the results to those obtained by qPCR.

**Results:**

We found that, with the exception of 25 bp reads, there is little difference for the detection of differential expression regardless of the read length. Once single-end reads are at a length of 50 bp, the results do not change substantially for any level up to, and including, 100 bp paired-end. However, splice junction detection significantly improves as the read length increases with 100 bp paired-end showing the best performance. We performed the same analysis on two ENCODE samples and found consistent results confirming that our conclusions have broad application.

**Conclusions:**

A researcher could save substantial resources by using 50 bp single-end reads for differential expression analysis instead of using longer reads. However, splicing detection is unquestionably improved by paired-end and longer reads. Therefore, an appropriate read length should be used based on the final goal of the study.

**Electronic supplementary material:**

The online version of this article (doi:10.1186/s13059-015-0697-y) contains supplementary material, which is available to authorized users.

## Background

One of the main questions for a researcher performing a sequencing experiment is the length of reads to use and whether to use single-end reads or paired-end reads. Longer reads should, a priori, increase the level of uniquely mapping reads, but such longer reads have an increased cost in reagents and an increase in running time for the instrument. While the determination of the proper read length for an experiment is important across all sequencing experiments, including genome re-sequencing, de novo sequencing, RNA-seq, and ChIP-seq, we have only focused on the use of RNA-seq for differentially expressed genes (DEGs) and isoform detection.

The initial reads on Illumina and other next-generation platforms were extremely short and often only ranged up to 25 or 36 bp [1]. While these reads were sufficient for some assays, a substantial percentage of the reads could not be mapped uniquely and were often discarded due to the inability to determine their correct matching location within the genome [2]. More recently, the lengths of reads have increased substantially and sequencers have been improved to allow for the sequencing of both ends of a fragment to allow for paired-end sequences. The current read length that is standard for many experiments is paired-end 100 bp reads and there is also the possibility of running paired-end 300 bp reads.

Since read lengths have increased substantially over recent years and will continue to increase, we decided to determine whether longer reads are more beneficial for RNA-seq DEG and isoform determination. Contrary to the assumption that substantial gains occur in the quality of the results as read length increases and when using paired ends, we found that, for DEGs, there is little improvement in the results as the length increased beyond 50 bp. Thus, a researcher can cut his or her sequencing budget by as much as half over 100 bp paired-end sequencing (Table 1). For isoform detection, however, we found strong evidence that longer reads are significantly better than shorter reads for the detection of both known and novel isoforms.

**Table 1:** Approximate cost of sequencing for each read length and sequencing type on a HiSeq 2500, high-output mode v3 (eight lanes per flowcell)

	Per lane cost	
Read configuration	Single end	Paired end
25 bp	$950	$1,275
50 bp	$1,100	$1,650
75 bp	$1,250	$2,025
100 bp	$1,400	$2,400

## Results

We have used data from the SEQC Sequencing study to investigate the effects of read-length on RNA-seq results and then validated the results using data from the ENCODE consortium. Since our main goal was to investigate the role of read length in determining RNA-seq results, we wanted to minimize all other variables. Therefore, we obtained the same sets of physical reads for the entire experiment and these reads were bioinformatically trimmed to produce reads of shorter lengths. This trimming is akin to what would have been obtained if the sequencing machine had been stopped earlier than it was for the longer reads. The quality and error profile of the 50^th^ base of a 50 bp read is the same as that of the 50^th^ base of a 100 bp read.

We took two samples from the Association of Molecular Resource Facilities (ABRF) SEQC study that consisted of RNA standards (see "Materials and methods"), labeled here as A and B. For each sample, we took three sets of paired-end 101 bp reads to form the basis of the analysis. These reads were then processed to produce 100, 75, 50 and 25 bp paired-end reads and 100, 75, 50 and 25 bp single-end reads. All of the reads were then aligned to the human genome using the STAR aligner.

### Mapping statistics and splice junction detection

**Fig. 1:**
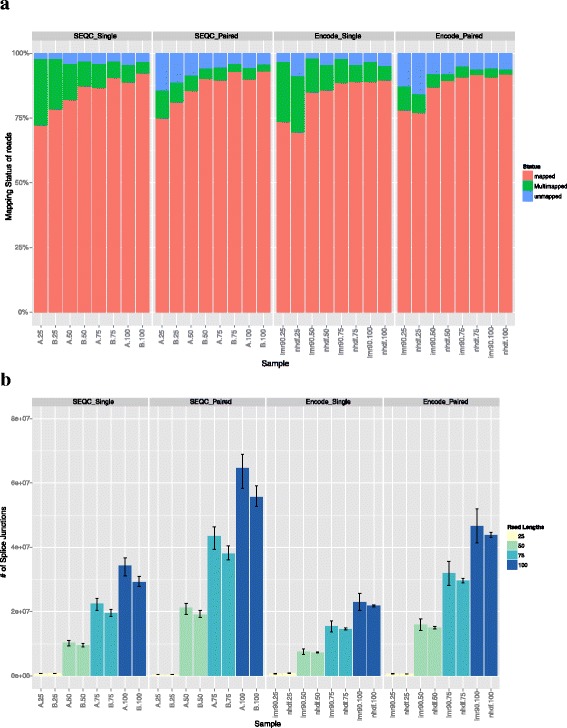
Mapping statistics of all samples and read lengths. **a** Each sample and read length is plotted with its respective percentage of uniquely mapped, multi-mapped and unmapped reads. The 25 bp reads have the lowest percent of uniquely mapped reads across all samples. Single-end reads also have higher percentage of multi-mapped reads and slightly lower percentage of uniquely mapped reads. **b** The number of splice junctions detected for each sample is plotted. The 25 bp samples detected the least number of junctions and single-end reads detected significantly fewer junctions overall than paired-end reads. The error bars represent the highest and lowest number of splice junctions detected across replicates

Mapping statistics were first examined (Fig. 1a), and the data showed overall consistent mapping statistics. All alignments of 25 bp read lengths contained a low number of uniquely mapped reads. This deficiency was partially improved when using 50 bp read lengths, while 75 bp and 100 bp reads contained almost the same number of uniquely mapped reads. The number of multi-mapped reads increased when using 25 bp reads, whereas all other read lengths were consistent. However, the number of multi-mapped reads increased significantly when using single-end reads (Fig. 1).

The number of splice junctions detected for each alignment was also determined (Fig. 1b). Alignments with 25 bp read lengths resulted in a significantly lower number of splice junctions detected (t = −13.13, *p* value = 2.2 × 10^−16^). Moreover, the numbers of splice junctions detected by the alignment of 100 bp reads was significantly higher than with any other read lengths (t = 7.08, *p* value = 9.5 × 10^−8^). Also, single-end reads detected fewer splice junctions overall when compared with the paired-end reads. In summary, longer paired-end reads are significantly better for splice junction detection.

### Differential expression at different read lengths

**Fig. 2:**
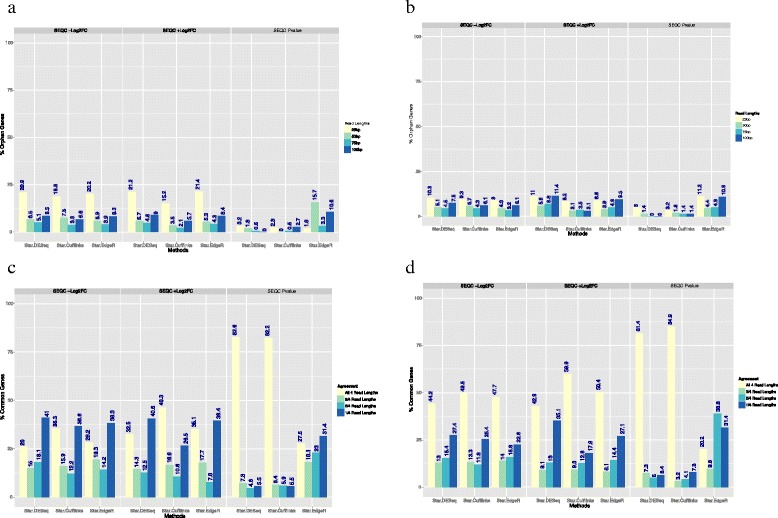
Determination of differentially expressed genes according to read length and differential expression method. **a** Single-end read samples. The number of orphan genes (read-length-specific genes) in the overlap of the top 200 genes sorted by -Log2-based fold change (-Log2FC; down-regulated), +Log2FC (up-regulated) and *p* value. **b** Paired-end read samples. The number of orphan genes (read-length-specific genes) in the overlap of the top 200 genes sorted by -Log2FC, +Log2FC and *p* value. **c** Single-end read samples. The plot shows the agreement for the top 200 differentially expressed genes by different read length. **d** Paired-end read samples. The plot shows the agreement for the top 200 differentially expressed genes by different read length

The aligned reads were processed through one of three computational pipelines to determine differential expression (DESeq, EdgeR and Cufflinks). The log_2_ fold-change data from these pipelines were used to determine DEGs and we extracted the top 200 DEGs from each pipeline for comparison. There was a high overall degree of consistency between the three pipelines (Fig. 2) for the sets of genes that were determined to be significantly differentially expressed for any specific read length.

The top 200 DEGs for all read lengths for each differential expression method and each sample were sorted both by log_2_ based fold-change (Log_2_FC) of both up-regulated (+Log2FC) and down-regulated genes (−Log2FC) and by *p* value and compared. The number of orphan (read-length-specific) genes was then calculated. For single-end samples (Fig. 2a), 25 bp read lengths gave the highest percentage (average of 13.8 %) of orphan genes among all differential expression methods and samples. This shows that the differential expression profile calculated using the 25 bp read length is significantly different. Using paired-end 25 bp reads reduced this difference (Fig. 2b; down to an average of 5 %); however, among paired-end reads, 25 bp reads still gave the highest difference. The differences between 50 bp, 75 bp and 100 bp, for both paired-end and single-end reads, were small (0–12 % orphan genes).

The number of read lengths that support any specific DEG was calculated using the same overlap as above. For single-end reads (Fig. 2c), the percentage of DEGs supported by all four read lengths is fairly low. This percentage is significantly higher (t = −4.85, *p* value = 0.00015) when using paired-end reads (Fig. 2d), which suggests that paired-end reads are better for determining DEGs. Also, sorting on *p* value instead of Log_2_FC improves the overlap when using single-end reads and significantly improves the overlap when using paired-end reads.

**Fig. 3:**
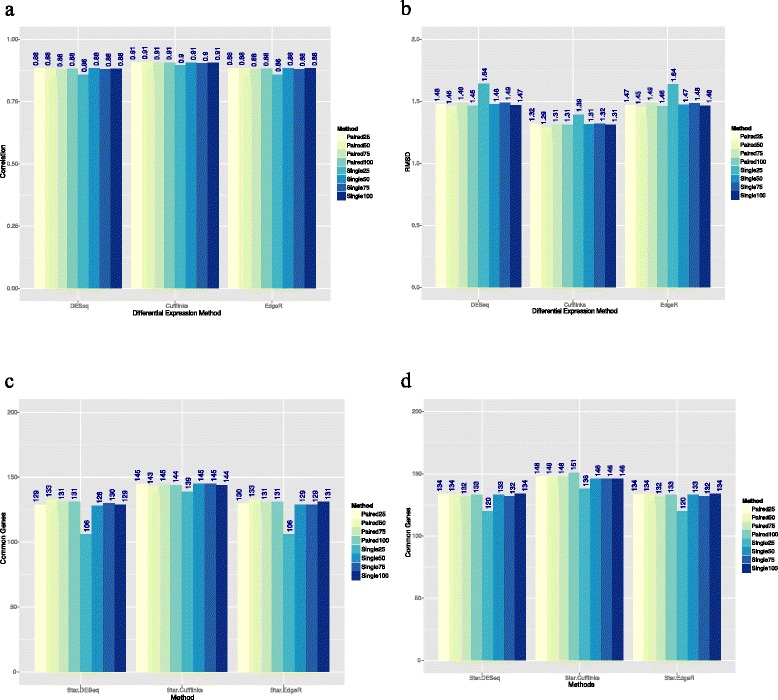
Comparison of previously reported qPCR results with our DEG results. **a** Pearson correlation between Log2FC of genes according to various differential expression methods and qPCR. Single-end 25 bp reads have the worst correlation when using DESeq and EdgeR. **b** Root mean square deviation (RMSD) between Log2FC and qPCR. Single-end 25 bp reads give results farthest from the true values. **c** Common genes between the top 200 genes identified by various differential expression methods and qPCR sorted by +Log2FC. **d** Same as (**c**), except sorted by –Log2FC. The overlap of common genes improves as read length increases. However, the gain is not significant for reads >50 bp for paired-end and >75 bp for single-end reads

For a true-positive comparison of gene expression, we used previously reported quantitative PCR (qPCR) results comparing the expression of samples A and B (Fig. 3). We calculated the Pearson correlation and root mean square deviation (RMSD) between all the DEGs for each read length and the qPCR results. While no read length results were completely concordant with the qPCR results, the lowest level of identity was achieved with the single-end and paired-end 25 bp reads and the quality did not significantly improve beyond that achieved with 50 bp single-end reads (Fig. 3a, b).

We next looked at the lists of the top 200 DEGs identified for each read length to determine their overlap (Fig. 3c, d). These lists represent the main result of an RNA-seq experiment performed using a particular read length. Overlap between the lists of the top 200 DEGs sorted by Log2FC shows that single-end 25 bp reads perform the worst; however, this performance is nicely recovered when using paired-end 25 bp reads (average of 121.5 versus 137 genes, respectively). Most 100 bp reads performed similarly when comparing paired-end versus single-end reads. Therefore, the gain in performance is not significant when using reads >50 bp for paired-end reads and >75 bp for single-end reads.

Additionally, we looked at genes that are paralogous (n = 21,459) to investigate whether longer read lengths result in higher mapping of overall reads to these genes (Figure S6a in Additional file 1). We calculated the total number of reads mapped to these genes in the paired-end SEQC dataset of samples A and B. To our surprise, with the exception of 25 bp reads, all the read lengths resulted in equal numbers of reads aligned to the paralogous genes. The overall trend of number of reads mapped as a function of read length stays the same regardless of paralogous gene length (Figure S6b in Additional file 1).

### Splicing detection

**Fig. 4:**
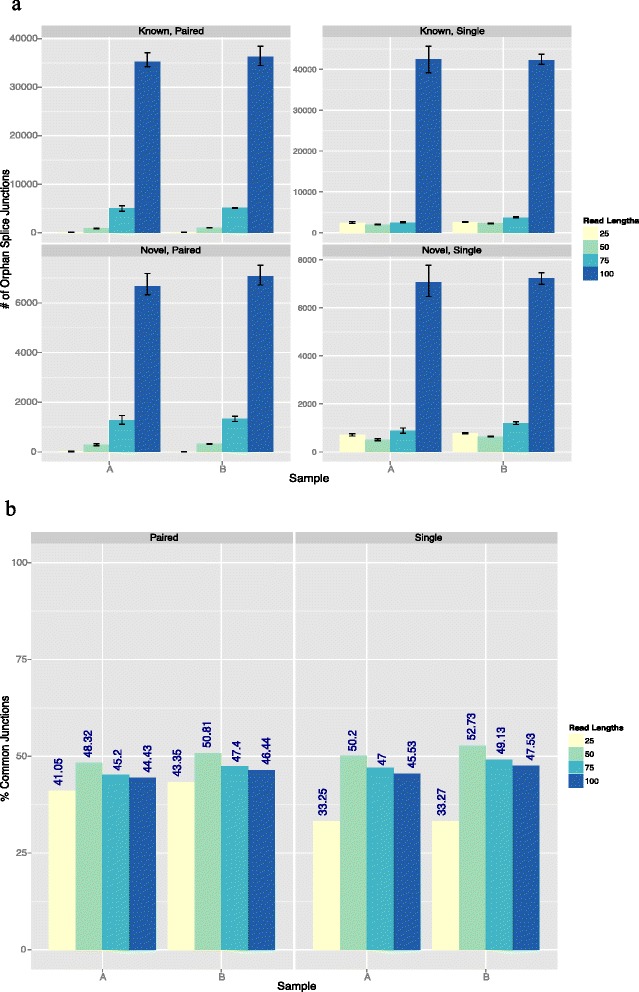
Splice junction agreement and inter-replicate reproducibility. **a** Number of known and novel junctions that were orphans (read-length-specific junctions) according to the read length in a specific sample. **b** Percentage of known junctions that were common when paired-end and single-end samples of the same read length were intersected. Error bars represent the range of all the replicates

Besides DEG detection, another function of RNA-seq is to determine splice sites and RNA isoforms. Using our variable length reads, we determined the number of splice sites that were detected using the STAR algorithm. We found that, for the detection of both known splice sites (Fig. 4a) and novel splice sites (Fig. 4b), there was a marked improvement with longer read lengths, and with the use of paired-end reads relative to single-end reads. The reason for this improvement is presumably because longer reads are generally superior for mapping and they have a greater chance of overlapping a splice junction, which is a major component of splicing detection. Based on the results, the longest reads (≥100 bp) should be used if junction detection is the primary goal of a sequencing experiment. This holds true for both known and novel junctions.

**Fig. 5:**
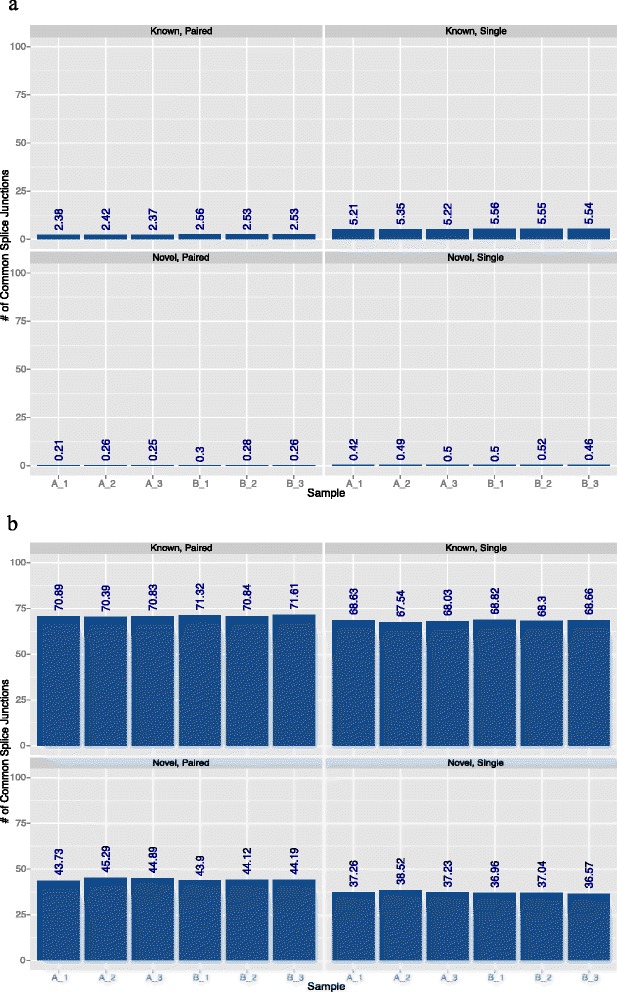
Common splice junctions detected with different read lengths. **a** Percentage of splice junctions detected with all four read lengths. **b** Percentage of splice junctions detected with all read lengths except 25 bp

The percentage of splice junctions that were detected with all the reads lengths was also determined (Fig. 5). Overall, the percentage of common splice junctions detected with all four read lengths is very small (Fig. 5a). All four read lengths detect only about 8–9 % of known splice junctions and about 1–2 % of novel splice junctions. However, since 25 bp read lengths contribute to many false positives, the overlap was also analyzed after discarding results found with the 25 bp read length (Fig. 5b). The percentage of common splice junctions increased to over 50 % for known splice junctions and over 20 % for novel junctions. This also shows that the 25 bp read length does not detect many splice junctions that are detected by reads lengths greater than 25 bp.

To further confirm this result, we examined well-validated, “golden junctions”, which were found by all five sequencing platforms from the ABRF study on RNA-seq [3] (Figure S1 in Additional file 1). These data showed that 25 bp reads showed the lowest percentage overlap with golden junctions (9.6 %). However, read lengths longer than 25 bp all showed high overlap (97 %) and there was relatively little difference between read lengths. Therefore, read lengths of 50 bp or longer can still be used for detecting bona fide junctions.

### Confirmation of results on ENCODE samples

In order to confirm our findings on an additional data set, we performed an identical analysis on RNA-seq data from NHDF and IMR90 cells obtained from an ENCODE study (Figures S3, S4, and S5 in Additional file 1). The only difference was that there were only two replicates available for each ENCODE sample whereas there were three replicates available for the SEQC samples. Additionally, there was no qPCR data available for the ENCODE samples. The results for SEQC samples were mainly replicated when using the ENCODE samples, with a consistent trend of little improvement over short reads’ utility for DEG detection but longer reads consistently improving isoform detection.

### Confirmation of results using RSEM/EBseq method

We also aligned all the SEQC samples using RSEM [4] and then performed differential expression using EBseq [5]. The differential expression tables were then analyzed to find the number of orphan reads and percentage overlap similar to in Fig. 2 (Figure S2 in Additional file 1). RSEM/EBseq showed higher overlap and less orphan genes between read lengths. However, the overall trend is still similar and thus the conclusions made using the STAR aligner still hold true.

## Discussion

When a researcher performs an RNA-seq experiment, he or she is confronted with the question of which read length to use and whether single-end reads are sufficient, or if they require the sequencing of both ends of their fragments. We have utilized two distinct datasets (SEQC and ENCODE) to provide insight into this critical aspect of experimental design. Our findings show that longer reads are not necessarily significantly better than shorter reads and an intermediate length of reads would provide adequate results for differential expression analysis.

We found that 25 bp reads are simply too short for effective use in most RNA-seq applications. They result in high numbers of multi-mapped reads and, depending upon the alignment protocol, may thus result in loss or inaccurate mapping of the data. This effect is even more pronounced when using 25 bp single-end reads. Also, they result in the highest number of orphan genes, which means the gene quantification accuracy may be questionable. Other than 25 bp reads, all other read lengths show similar accuracy and there is little gain in accuracy with longer read lengths. Paired-end reads improve accuracy of differential expression as shown by the qPCR comparison, although this gain is not significant. Paired-end reads are useful more at the alignment level than the differential expression level. They lower the percentage of multi-mapped reads and thus fewer reads are thrown out before quantification. They might also help in quantifying genes that have a high number of duplicated regions, as evidenced by our analysis of pseudogenes.

For an experiment whose goal is only to determine differential expression, the improvement beyond 50 bp single-end reads is not substantial and would not justify the added cost. This lack of improvement is due to the fact that there is not a considerable gain in the mappability of sequencing reads beyond 50 bp read lengths. This mapping lies at the heart of detecting differential expression when the reads from two conditions are mapped back to the reference genome and the number of reads mapping to a gene in each condition are compared (after normalization). Using 50 bp single-end reads costs half as much as using 100 bp paired-end reads (Table 1). Thus, the amount of money saved is substantial.

More troubling, though, is the lack of consistency of results between different read lengths. One would hope that genes that were detected as differentially expressed at one read length would also be detected as differentially expressed at a different read length since the experiment properly represented the expression of genes in the cell. We found this not to be the case and there was substantial variability in the lists of top DEGs between read lengths. This is likely due to a combination of splice junction overlap and gene annotation (e.g., short genes), as well as the usual factors inherent to library preparation, such as library size, RNA fragmentation, and GC content biases [6].

For splicing, we found contrasting results to those for DEGs. Since splicing detection inherently relies on sequence assembly, and longer sequences improve assembly, longer sequences led to superior splicing detection. This improvement was for the detection of both known and novel splice variants. Use of 100 bp reads not only resulted in many more splice junctions being detected than with smaller read lengths, but also the detection of many splice junctions that are specific to 100 bp reads only. These junctions would not have been detected if smaller read lengths were used. The percentage of common splice junctions between all four read lengths is very low, but improves if 25 bp read lengths are not considered. This suggests that 25 bp reads do not find most of the splice junctions detected by longer reads.

## Conclusions

The answer for the ideal length of sequencing for RNA-seq depends on the desired results of the experiment. If only a list of DEGs is desired, then 50 bp single-end reads would be sufficient for most studies. In contrast, for splicing detection, our results suggest that the longest reads possible should be used, including using paired-end reads.

## Materials and methods

### RNA-seq samples

The raw data for this analysis came from two samples from the ABRF SEQC study. The Universal Human Reference RNA (740000, Agilent Technologies) and Ambion FirstChoice Human Brain Reference RNA (AM6000, Life Technologies) were used as samples A and B, respectively, in the MicroArray Quality Control (MAQC) experiments initiated in 2005 and summarized in *Nature Biotechnology* in 2006 [7]. These RNA samples are well characterized and were used as part of the SEQC study by the US Food and Drug Administration [Seqc/Maqc-III Consortium. "A comprehensive assessment of RNA-seq accuracy, reproducibility and information content by the Sequencing Quality Control Consortium." *Nature biotechnology* 32.9 (2014): 903-914.]

Three paired-end 100 bp replicates of sample A and three paired-end 100 bp replicates of sample B were selected and downloaded (Gene Expression Omnibus accession GSE47792). For confirmation of our results in an independent sample, we used two replicates each of paired-end 100 bp ENCODE RNA-seq samples for IMR90 and NHDF cells. The files were downloaded from the UCSC ENCODE repository [8] with sample names Nhdf70717012CellTotalFastqRd[1, 2]Rep1, Nhdf70717012CellTotalFastqRd[1, 2]Rep2, Imr90CellTotalFastqRd[1, 2]Rep1, and Imr90CellTotalFastqRd[1, 2]Rep2.

### Sequencing data preprocessing

FASTQC [9] was run on all the samples to make sure there were no sequencing errors. Raw sequences were aligned to hg19 genome assembly (UCSC) with STAR RNA-seq aligner version 2.3.0e [10]. In order to eliminate variability between sequencing runs, we used the same exact reads and trimmed them to lengths of 100, 75, 50 and 25 bp computationally and also separated the paired ends to only use the first read from each pair in order to simulate single-end sequencing.

### RNA-seq differential gene expression analysis

Read counts were calculated using the BedTools’ intersectBed command [11] and the GENCODE v.17 annotation was used for gene calls. Sample A versus sample B and imr90 versus nhdf differential expression were run on all read counts with single-end and paired-end reads. Three methods of differential expression were used: Cufflinks v.2.1.1 [12], DESeq v.1.10.1 [13] and EdgeR v.3.0.8 [14]. Aligned BAM files were provided to Cuffdiff from the Cufflinks suite with default options. Raw read counts of uniquely aligned reads were used for DESeq and EdgeR. All read counts where the average read count from all three replicates was less than 10 were discarded before running analysis with DESeq and EdgeR. The differential expression tables were then analyzed using *p* values and log2FC cutoffs. This analysis was also performed using the aligner RSEM [4] and differential expression method EBseq [5]. The gene IDs for paralogous genes were downloaded from Ensembl Biomart v.72 by selecting attributes, then homology, and then paralogs. The list was further filtered by homology type to only keep within_species_paralog.

### PrimePCR RT-qPCR gene expression analysis

RT-qPCR data were only available for samples A and B. Undetectable C_q_ values (C_q_ > 35 or C_q_ = 0) were removed from data for samples A and B. The standard deviation of the C_q_ values for each gene was calculated, and the gene MYSM1 exhibited the lowest standard deviation. The data were normalized by subtracting the average Cq of MYSM1 from each PrimePCR target to give the log_2_ difference between the endogenous control and the target gene. The normalized Cq values were used to calculate Log_2_FC between A and B. This differential expression Log_2_FC was then used to calculate the Pearson and Spearman correlation to the RNAseq data using the cor() function from the R stats package. The RMSD was also calculated using the Log_2_FC of RNAseq and qPCR data.

### Splice junction analysis

Non-canonical junction detection was turned off in order for the aligned files to be compatible with Cufflinks. All other parameters were left as default. Splice junction files produced by STAR were used for all splice junction analysis. The total number of splice junctions detected for each sample and read length was taken from the log.final.out file printed by the aligner STAR [10]. SJ.out.tab is also a file printed by STAR, which contains all the junctions detected, and other metrics for each junction. STAR was run in novel splice junction detection mode and there was no splice junction database provided. These junctions were intersected using bedtools [11] and the Venn diagrams were created using VennDiagram package in R [15]. Known junctions were annotated using 1 bp window of GENCODE v.17 splice junction file since STAR output is 1-based and GENCODE bed files are 0-based. Overlap plots were created using ggplot2 [16]. The “golden junctions” are junctions found by all five platforms (454, Illumina, PGM, Proton, 454) [3].

## Additional file

Additional file 1: Supplementary Figures. A figure that shows the percent of “golden junctions” detected in all current NGS platforms (454, Illumina, PGM, Proton, 454) when intersected with each sample of different read lengths. **Supplementary Table.** The supplementary table contains the numerical data for all main text figures (Fig 1 – Fig 5). Each sheet in the file contains data for part of figure as referred by sheet name.

## Abbreviations

ABRF: Association of Molecular Resource Facilities
bp: base pair
DEG: differentially expressed gene
Log2FC: log2-based fold change
qPCR: quantitative PCR
RMSD: root mean square deviation.
