# Supplementary material for: The impact of read length on quantification of differentially expressed genes and splice junction detection
Source: Genome Biol. 2015 Jun 23;16(1):131. doi: 10.1186/s13059-015-0697-y (PMC4531809; doi:10.1186/s13059-015-0697-y)
Supplement: Additional file 1: Supplementary Figures. — A figure that shows the percent of “golden junctions” detected in all current NGS platforms (454, Illumina, PGM, Proton, 454) when intersected with each sample of different read lengths. Supplementary Table. The supplementary table contains the numerical data for all main text figures (Fig 1 – Fig 5). Each sheet in the file contains data for part of figure as referred by sheet name. [file 13059_2015_697_MOESM1_ESM.zip › Supplementary Figures.docx]

**Supplementary Figures**

**Fig S1. Percent of Golden Junctions detected.** The Figure shows the mean percent of golden junctions detected across replicates when intersected with each sample of different read lengths.

| a.   | b.   |
| --- | --- |
| c.   | d.   |

**Fig. S2. Agreement of Differential Expression Genes between read Lengths and Differential expression Methods (including RSEM) on SEQC Samples**. (a) Single-end read samples. Number of Orphan genes (Read-length specific genes) in the overlap of top 200 genes sorted by -Log2FC (down-regulated), +Log2FC (up-regulated) and P-value. (b) Paired-end read samples. Number of Orphan genes (Read-length specific genes) in the overlap of top 200 genes sorted by -Log2FC, +Log2FC and P-value. (c) Single-end read samples. Plot shows the agreement on top 200 differentially expressed genes by different read lengths. (d) Paired-end read samples. Plot shows the agreement on top 200 differentially expressed genes by different read lengths.

| a. | b.   |
| --- | --- |
| c.   | d.   |

**Fig. S3. Agreement of differential expression genes between read lengths and differential expression methods for encode Samples**. (a) Single-end read samples. Number of Orphan genes (Read-length specific genes) in the overlap of top 200 genes sorted by -Log2FC (down-regulated), +Log2FC (up-regulated) and P-value. (b) Paired-end read samples. Number of Orphan genes (Read-length specific genes) in the overlap of top 200 genes sorted by -Log2FC, +Log2FC and P-value. (c) Single-end read samples. Plot shows the agreement on top 200 differentially expressed genes by different read lengths. (d) Paired-end read samples. Plot shows the agreement on top 200 differentially expressed genes by different read lengths.

| a.   |
| --- |
| b.   |

**Fig S4. Splice Junctions Agreement and inter-replicate reproducibility for Encode samples.** (a). Number of known and novel junctions that were orphans (Read-Length specific junctions) when compared with all the read-lengths of specific sample. (b). Percent of known junctions that were common when paired-end and single-end samples of the same read-length were intersected. Error bars represent the range of all the replicates.

| a.   |
| --- |
| b.   |

**Fig S5. Common Splice Junctions between Read Lengths for encode samples.** (a) Percent of splice junctions detected by all 4 read lengths. (b) Percent of splice junctions detected by all read lengths except 25bps.

| a. |
| --- |
| b. |

**Fig S6.** **Alignment of reads to paralogous genes**. (a) The plot shows aligned normalized read counts for each read length and sample to within-species-paralogous genes as computed by Ensembl. (b) Aligned read counts for each read length sub-divided by median paralogous gene length (2440bp).

| a.  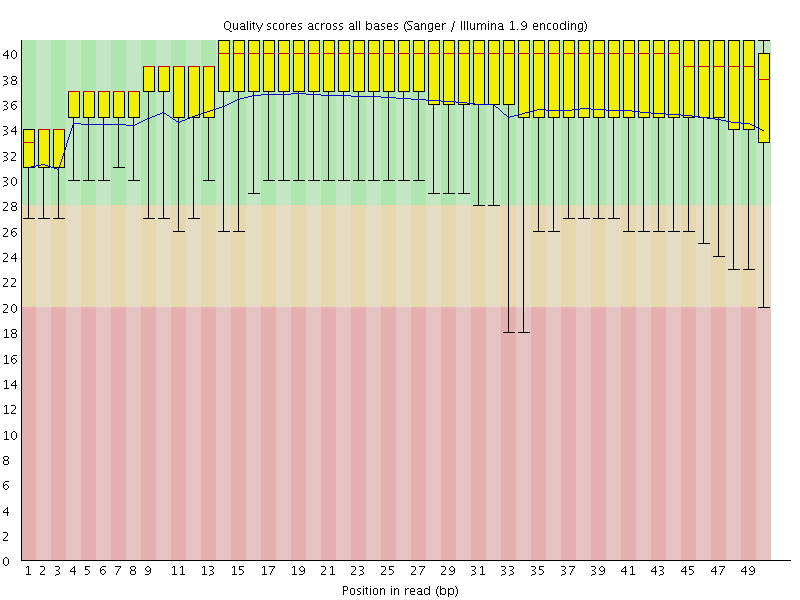 |
| --- |
| b.  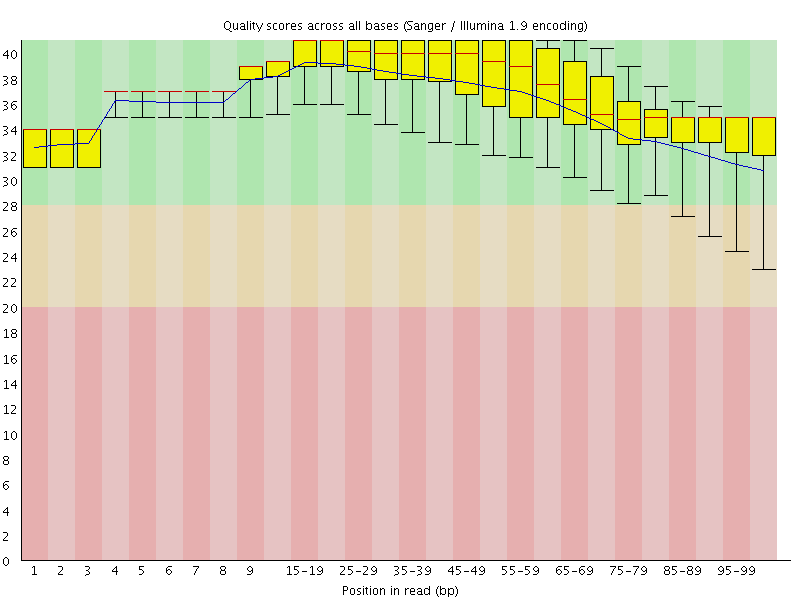 |

**Fig S7. Per Base Phred Quality plots for 50bp reads and 100bp reads.** These quality plots are created from untrimmed reads of Sample A as produced by the sequencer. (a) 50bp reads. (b) 100bp reads.

**Figure S8. Venn Diagram of all read lengths of top 200 DE genes in SEQC paired-end samples using Cufflinks sorted by –Log2FC.**

**Figure S9. Venn Diagram of all read lengths of top 200 DE genes in SEQC paired-end samples using Cufflinks sorted by +Log2FC.**

**Figure S10. Venn Diagram of all read lengths of top 200 DE genes in SEQC paired-end samples using Cufflinks sorted by Pvalue.**

**Figure S11. Venn Diagram of all read lengths of top 200 DE genes in SEQC single–end samples using Cufflinks sorted by –Log2FC.**

**Figure S12. Venn Diagram of all read lengths of top 200 DE genes in SEQC single–end samples using Cufflinks sorted by +Log2FC.**

**Figure S13. Venn Diagram of all read lengths of top 200 DE genes in SEQC single–end samples using Cufflinks sorted by Pvalue.**

**Figure S14. Venn Diagram of all read lengths of top 200 DE genes in SEQC paired-end samples using DESeq sorted by –Log2FC.**

**Figure S15. Venn Diagram of all read lengths of top 200 DE genes in SEQC paired-end samples using DESeq sorted by +Log2FC.**

**Figure S16. Venn Diagram of all read lengths of top 200 DE genes in SEQC paired-end samples using DESeq sorted by Pvalue.**

**Figure S17. Venn Diagram of all read lengths of top 200 DE genes in SEQC single–end samples using DESeq sorted by –Log2FC.**

**Figure S18. Venn Diagram of all read lengths of top 200 DE genes in SEQC single–end samples using DESeq sorted by +Log2FC.**

**Figure S19. Venn Diagram of all read lengths of top 200 DE genes in SEQC single–end samples using DESeq sorted by Pvalue.**

**Figure S20. Venn Diagram of all read lengths of top 200 DE genes in SEQC paired-end samples using EdgeR sorted by –Log2FC.**

**Figure S21. Venn Diagram of all read lengths of top 200 DE genes in SEQC paired-end samples using EdgeR sorted by +Log2FC.**

**Figure S22. Venn Diagram of all read lengths of top 200 DE genes in SEQC paired-end samples using EdgeR sorted by Pvalue.**

**Figure S23. Venn Diagram of all read lengths of top 200 DE genes in SEQC single–end samples using EdgeR sorted by –Log2FC.**

**Figure S24. Venn Diagram of all read lengths of top 200 DE genes in SEQC single–end samples using EdgeR sorted by +Log2FC.**

**Figure S25. Venn Diagram of all read lengths of top 200 DE genes in SEQC single–end samples using EdgeR sorted by Pvalue.**

**Figure S26. Venn Diagram of all read lengths of top 200 DE genes in ENCODE paired-end samples using Cufflinks sorted by –Log2FC.**

**Figure S27. Venn Diagram of all read lengths of top 200 DE genes in ENCODE paired-end samples using Cufflinks sorted by +Log2FC.**

**Figure S28. Venn Diagram of all read lengths of top 200 DE genes in ENCODE paired-end samples using Cufflinks sorted by Pvalue.**

**Figure S29. Venn Diagram of all read lengths of top 200 DE genes in ENCODE single–end samples using Cufflinks sorted by –Log2FC.**

**Figure S30. Venn Diagram of all read lengths of top 200 DE genes in ENCODE single–end samples using Cufflinks sorted by +Log2FC.**

**Figure S31. Venn Diagram of all read lengths of top 200 DE genes in ENCODE single–end samples using Cufflinks sorted by Pvalue.**

**Figure S32. Venn Diagram of all read lengths of top 200 DE genes in ENCODE paired-end samples using DESeq sorted by –Log2FC.**

**Figure S33. Venn Diagram of all read lengths of top 200 DE genes in ENCODE paired-end samples using DESeq sorted by +Log2FC.**

**Figure S34. Venn Diagram of all read lengths of top 200 DE genes in ENCODE paired-end samples using DESeq sorted by Pvalue.**

**Figure S35. Venn Diagram of all read lengths of top 200 DE genes in ENCODE single–end samples using DESeq sorted by –Log2FC.**

**Figure S36. Venn Diagram of all read lengths of top 200 DE genes in ENCODE single–end samples using DESeq sorted by +Log2FC.**

**Figure S37. Venn Diagram of all read lengths of top 200 DE genes in ENCODE single–end samples using DESeq sorted by Pvalue.**

**Figure S38. Venn Diagram of all read lengths of top 200 DE genes in ENCODE paired-end samples using EdgeR sorted by –Log2FC.**

**Figure S39. Venn Diagram of all read lengths of top 200 DE genes in ENCODE paired-end samples using EdgeR sorted by +Log2FC.**

**Figure S40. Venn Diagram of all read lengths of top 200 DE genes in ENCODE paired-end samples using EdgeR sorted by Pvalue.**

**Figure S41. Venn Diagram of all read lengths of top 200 DE genes in ENCODE single–end samples using EdgeR sorted by –Log2FC.**

**Figure S42. Venn Diagram of all read lengths of top 200 DE genes in ENCODE single–end samples using EdgeR sorted by +Log2FC.**

**Figure S43. Venn Diagram of all read lengths of top 200 DE genes in ENCODE single–end samples using EdgeR sorted by Pvalue.**
